# Human Papillomavirus Vaccine Course Completion Among Gay and Bisexual Men Who Have Sex With Men From a Time-Limited HPV Vaccination Catch-Up Program in Victoria, Australia

**DOI:** 10.3389/fpubh.2021.754112

**Published:** 2021-10-07

**Authors:** Kay Htaik, Christopher K. Fairley, Marcus Y. Chen, Rebecca Wigan, Elena Rodriguez, Catriona S. Bradshaw, Eric P. F. Chow

**Affiliations:** ^1^Melbourne Sexual Health Centre, Alfred Health, Melbourne, VIC, Australia; ^2^Central Clinical School, Faculty of Medicine, Nursing and Health Sciences, Monash University, Melbourne, VIC, Australia; ^3^Centre for Epidemiology and Biostatistics, Melbourne School of Population and Global Health, The University of Melbourne, Melbourne, VIC, Australia

**Keywords:** HPV, vaccination, men, cancer, genital warts, prevention, anal warts, immunisation

## Abstract

**Background:** To examine completion of the human papillomavirus (HPV) vaccination 3-dose regimen and factors associated with completion among men who have sex with men (MSM) aged ≤ 26 years participating in a time-limited HPV catch-up vaccination program in Victoria, Australia.

**Methods:** MSM who received their first dose of HPV vaccine at Melbourne Sexual Health Centre in 2017 were followed until October 2019. Vaccination completion was defined as those who received three doses. Multivariable logistic regression was performed to examine factors associated with vaccine completion.

**Results:** 931 of 1,947 (47.8%) eligible men received at least one dose of HPV vaccine, 750 (38.5%) received two and 590 (30.3%) received three doses. The median time to receiving the second and third dose was 2.8 (IQR = 2.1–4.8) and 7.2 (IQR = 6.3–10.7) months, respectively. Gay men had higher odds of receiving three doses compared to bisexual men (aOR = 2.17; 95%CI: 1.16–4.04). Compared with HIV-negative MSM not taking PrEP, HIV-positive MSM were more like to complete vaccination (aOR = 3.92, 95%CI: 1.62–9.47) but no difference was found compared to HIV-negative men taking PrEP (aOR = 1.55; 95%CI: 0.95–2.53).

**Conclusion:** Less than one-third of men aged ≤ 26 years completed the three doses of HPV vaccine. Further studies are needed to understand the barriers of men not completing the vaccine.

## Introduction

Human papillomavirus (HPV) is one of the most common sexually transmitted viral infections and is associated with genital warts, and cervical, penile and anal cancers. Multiple countries have introduced HPV vaccination programs for girls and women since 2006. A few countries have also introduced gender-neutral programs to include boys (1–3). Gay, bisexual and other men who have sex with men (MSM) are less likely to receive herd protection from female-only HPV vaccination programs. Given MSM have a high HPV prevalence and are at risk for anal cancer (4–7), some countries such as the UK, the US, Canada (i.e. only some provinces), Australia (i.e. only some states), have introduced targeted HPV vaccination programs for MSM. Past studies have shown that the initiation of HPV vaccine among adult MSM varied between settings, for instance, 25.8–34.8% in Canada (2017–2019) (8), and 42.6% in Australia (2017) (9). However, there have been few studies examining the proportion of MSM who completed the 3-dose course HPV vaccination from these MSM programs.

Australia has had a school-based vaccination program for girls aged 12–13 years since 2007 and this program was expanded to include boys aged 12–13 years in 2013. Between April 2017 and October 2019, the Victorian Government funded and implemented a targeted catch-up 3-dose quadrivalent HPV vaccination program targeting MSM aged ≤ 26 years through sexual health and primary care clinics (10). The recommended regime of the 3-dose HPV vaccine was 0, 1–2 and 6 months (11). We have previously shown that 42.6% of MSM attendees aged 16–26 years received their first dose of HPV vaccine at the Melbourne Sexual Health Centre (MSHC), Australia, in 2017. The study aimed to examine the proportion of MSM in this cohort who completed the three doses of the HPV vaccine and the factors associated with completing the full course of the HPV vaccination.

## Methods

This was a retrospective cohort study of MSM who received the first dose of HPV vaccine at the MSHC in 2017 (between April and December 2017). MSHC is the largest public HIV/STI clinic in Victoria where men can access HIV/STI services free of charge. HPV vaccines were provided to all MSM aged 16–26 years at MSHC for free between April 2017 and October 2019 during the time-limited HPV vaccination program funded by the Victoria Government.

There were 1,947 MSM aged 16–26 years who attended MSHC between April and December 2017 and reported that they did not receive any HPV vaccines from school. These men were eligible for the catch-up program, 830 men (43%) received the first dose of the vaccine at their first consultation during 2017, and the results are described elsewhere (9). Men were given a card with a reminder of a second and third appointments for their HPV vaccines after they received the first dose. In the present study, we analysed data from these 1,947 eligible men. We defined the vaccine completion as men who received the first dose of HPV vaccine at any time point at MSHC between April and December 2017, this included men who did not receive the vaccine at the first consultation but received the vaccine at any subsequent clinic visit throughout 2017. All men were followed until October 2019, the time when the catch-up program was ceased. Two investigators (KH and RW) reviewed the medical files of all 1,947 MSM to verify whether the individuals had received the vaccine; if so, the number of doses and the date of each dose were extracted.

We defined vaccination completion as individuals who received three doses of HPV vaccine given at MSHC. We reported the proportion of men who received one, two and three doses of vaccine. The 95% confidence intervals (CI) for the proportion was calculated using the binomial exact method. We calculated the median time from the first to second, and from the first to third doses and presented in months. Demographic characteristics such as age, country of origin, indigenous status, HIV status, use of pre-exposure prophylaxis (PrEP), condoms use and the number of partners in the past 12 months were also extracted from the patient's electronic medical records. Univariable and multivariable logistic regression was performed to examine factors associated with HPV vaccine completion among men who had received at least one dose of vaccine. Variables with *p* < 0.10 in the univariable analyses were considered as potential confounding factors and were included in the multivariable analyses. Crude and adjusted odds ratio (OR) and the corresponding 95% CIs were reported. All data analyses were conducted in Stata (version 17). This study was approved by the Alfred Hospital Ethics Committee, Melbourne, Australia (706/19).

## Results

**Figure 1:**
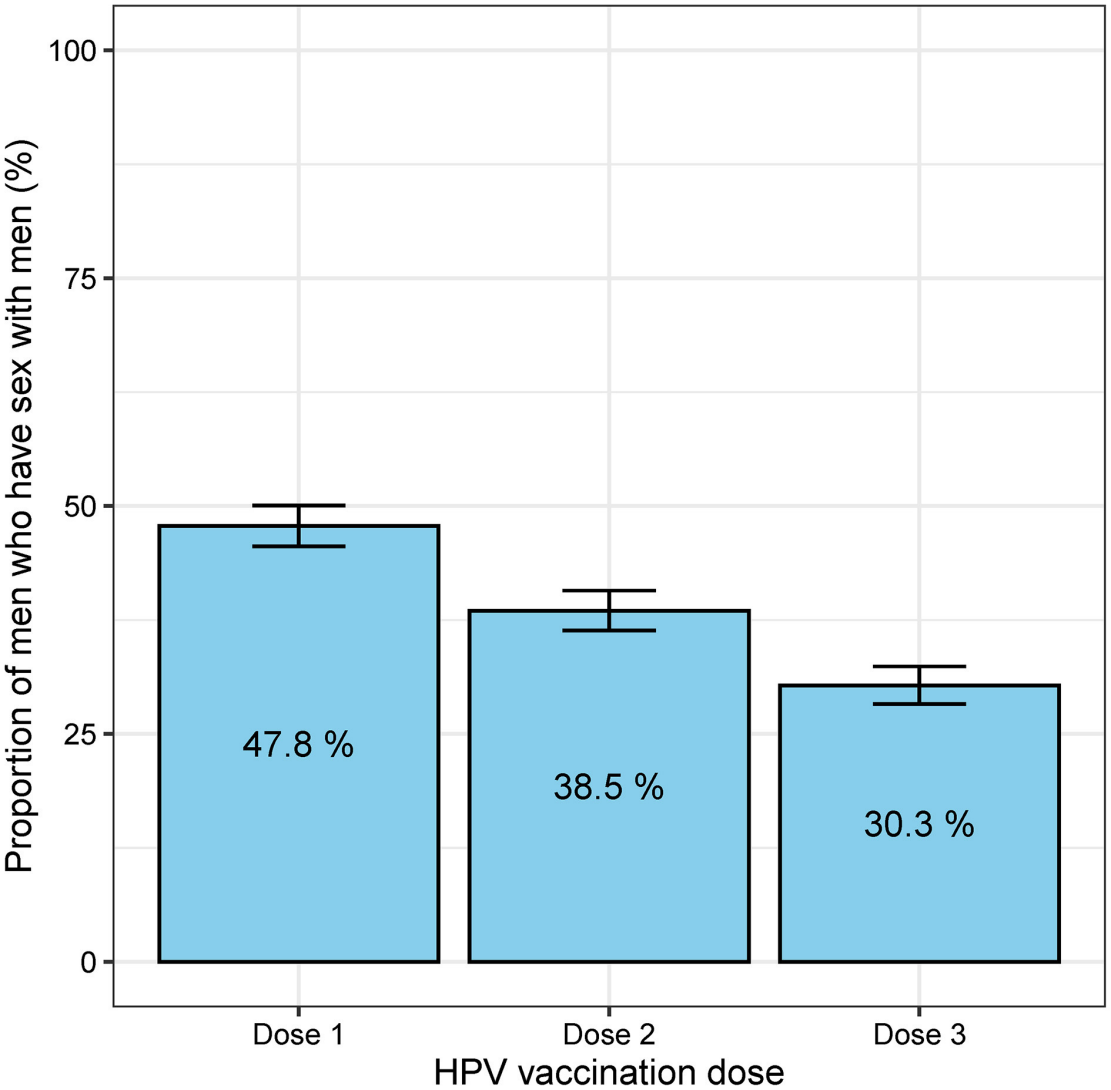
The proportion of 1,947 men who have sex with men who received the first, second and third doses of HPV vaccine. *The error bars represent the 95% confidence intervals of the proportion*.

**Figure 2:**
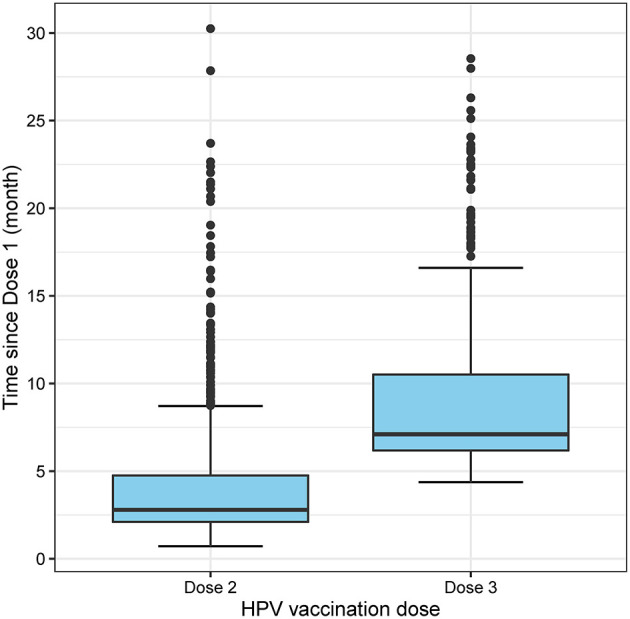
Boxplot showing the time of receiving the second and third dose from the first dose of HPV vaccine.

Between April and December 2017, 1,947 MSM aged 16–26 years attended MSHC and reported that they did not receive any HPV vaccine at school. Table 1 shows the majority of men aged 20–26 years (*n* = 1,843, 94.7%) and were born in Australia (*n* = 1,013, 52.0%). A small proportion of men reported having sex with both men and women (*n* = 166, 8.5%). There were 1,735 (89.1%) HIV-negative men not taking PrEP, followed by 144 (7.4%) HIV-negative men taking PrEP, and 68 (3.5%) men living with HIV. Of the 1,947 men, 931 (47.8%) received at least one dose of HPV vaccine in 2017 including 590 (30.3%) men who received three doses, 160 men (8.2%) received two doses only and 181 men (9.3%) received one dose only (Figure 1). Additionally, of the 931 who at least one dose of HPV vaccine, 590 (63.4%) men received all three doses. After completing the first dose, the median time to receiving the second dose was 2.8 (IQR 2.1–4.8) months and the third dose was 7.2 (IQR 6.3–10.7) months (Figure 2).

**Table 1:** Characteristics of 1,947 men who have sex with men aged 16–26 years attending the Melbourne Sexual Health Centre between April and December 2017.

**Characteristics**	**Number of men, *n***	**Proportion of men, *%***
**Age**		
16–19	104	5.3%
20–26	1,843	94.7%
**Country of birth**		
Australia	1,013	52.0%
Overseas	889	45.7%
Unknown/missing	45	2.3%
**Indigenous origin**		
Indigenous origin	29	1.5%
Non-Indigenous origin	1,726	88.6%
Unknown/missing	192	9.9%
**Past genital warts**		
No	1,842	94.6%
Yes	105	5.4%
Sexual orientation^*^		
Bisexual	166	8.5%
Gay	1,781	91.5%
**Number of male partners in the past 12 months^†^**		
0–4	755	38.8%
≥5	863	44.3%
Unknown/missing	329	16.9%
**Condom use with male partners in the past 12 months**		
Always	438	22.5%
Not always	1,055	54.2%
Unknown/missing	454	23.3%
**Current sex worker**		
No	1,619	83.2%
Yes	15	0.8%
Unknown/missing	313	16.1%
**HIV status and PrEP use**		
HIV negative, not taking PrEP	1,735	89.1%
HIV negative, taking PrEP	144	7.4%
HIV positive	68	3.5%

* *Sex orientation was based on self-reported sexual practices in the past 12 months. Gay was defined as men who have sex with men only; while bisexual was defined as men who have sex with men and women*.

† *The number of male partners in the past 12 months was categorised using the median*.

The proportion of men who received three doses of HPV vaccine varied by HIV status and PrEP use. Table 2 shows that more HIV-positive men completed three doses of vaccine (89.7%, 52/58), followed by HIV-negative men taking PrEP (70.2%, 66/94) and HIV-negative men not taking PrEP (60.6%, 472/779). Compared to HIV-negative men not taking PrEP, HIV-positive men had 3.92 (95% CI 1.62–9.47) times higher odds of receiving three doses of HPV vaccine, after adjusting other potential confounder factors (Table 2). Furthermore, gay men (i.e., men who had sex with men only) had 2.17 (1.16–4.04) times higher odds of receiving three doses compared to bisexual men (i.e., men who had sex with men and women). However, HPV vaccine completion was not significantly associated with sexual practices, past history of genital warts, age, country of birth and the travel distance from residential address to the clinic.

**Table 2:** Factors associated with completing three doses of HPV vaccinations in 931 men who had received at least one dose of vaccine.

**Factors**	***n*** **/*N* (%)**	**Crude OR (95% CI)**	***P*** **-value**	**Adjusted OR (95% CI)**	***P*** **-value**
**Age**					
16–19	24/38 (63.2%)	1 (ref)			
20–26	566/893 (63.4%)	1.01 (0.52–1.98)	0.978		
**Country of birth**					
Australia	302/502 (60.2%)	1 (ref)		1 (ref)	
Overseas	271/409 (66.3%)	1.30 (0.99–1.71)	0.058	1.27 (0.96–1.69)	0.099
Unknown/missing	17/20 (85.0%)	3.75 (1.09–12.97)	0.037	2.92 (0.83–10.34)	0.096
**Indigenous origin**					
Indigenous origin	9/14 (64.3%)	1 (ref)			
Non-Indigenous origin	521/832 (62.6%)	1.07 (0.36–3.24)	0.898		
Unknown/missing	60/85 (70.6%)	1.43 (0.88–2.33)	0.148		
Travel distance from residual address to the clinic (km)^*^					
0–5	199/301 (66.1%)	1 (ref)			
6–10	124/185 (67.0%)	1.04 (0.71–1.54)	0.836		
11–15	50/82 (61.0%)	0.80 (0.48–1.33)	0.388		
16–20	37/58 (63.8%)	0.90 (0.50–1.62)	0.733		
≥21	91/142 (64.1%)	0.91 (0.60–1.39)	0.675		
Unknown/missing	89/163 (54.6%)	0.62 (0.42–0.91)	0.015		
**Past genital warts**					
No	552/869 (63.5%)	1 (ref)			
Yes	38/62 (61.3%)	0.91 (0.54–1.54)	0.725		
**Sexual orientation^†^**					
Bisexual	18/45 (40.0%)	1 (ref)		1 (ref)	
Gay	572/886 (64.6%)	2.73 (1.48–5.04)	0.001	2.17 (1.16–4.04)	0.015
**Number of male partners in the past 12 months^‡^**					
0–4	170/292 (58.2%)	1 (ref)		1 (ref)	
≥5	249/419 (59.4%)	1.05 (0.78–1.42)	0.747	1.01 (0.73–1.40)	0.935
Unknown/missing	171/220 (77.7%)	2.50 (1.69–3.71)	<0.001	2.17 (0.61–7.69)	0.230
**Condom use with male partners in the past 12 months**					
Always	107/173 (61.8%)	1 (ref)		1 (ref)	
Not always	291/499 (58.3%)	0.86 (0.61–1.23)	0.415	0.81 (0.56–1.17)	0.266
Unknown/missing	192/259 (74.1%)	1.77 (1.17–2.67)	0.007	0.67 (0.32–1.38)	0.274
**Current sex worker**					
No	417/704 (59.2%)	1 (ref)		1 (ref)	
Yes	5/11 (45.5%)	0.57 (0.17–1.90)	0.362	0.49 (0.14–1.71)	0.263
Unknown/missing	168/216 (77.8%)	2.41 (1.69–3.43)	<0.001	1.14 (0.35–3.69)	0.825
**HIV status and PrEP use**					
HIV negative, not taking PrEP	472/779 (60.6%)	1 (ref)		1 (ref)	
HIV negative, taking PrEP	66/94 (70.2%)	1.53 (0.96–2.44)	0.072	1.55 (0.95–2.53)	0.082
HIV positive	52/58 (89.7%)	5.64 (2.39–13.28)	<0.001	3.92 (1.62–9.47)	0.002

* *The travel distance between the residual's postcode and the clinic's postcode was measured by land transport. Individuals who lived in interstate were categorised into the “unknown/missing” group*.

† *Sex orientation was based on self-reported sexual practices in the past 12 months. Gay was defined as men who have sex with men only; while bisexual was defined as men who have sex with men and women*.

‡ *The number of male partners in the past 12 months was categorised using the median*.

## Discussion

A targeted time-limited catch-up HPV vaccination program for MSM aged ≤ 26 years was introduced in Victoria, Australia, between April 2017 and October 2019. In a sexual health clinic setting, we found that less than half of eligible MSM received at least one dose of the vaccine in 2017 and less than one-third of men received all three doses. The HPV vaccine uptake and completion was low among young MSM although the HPV vaccines were provided for free via a catch-up program. The median time of receiving doses 2 and 3 was at 2.8 and 7.2 months, respectively, these intervals were similar to the recommended intervals between doses (i.e., at 1–2 and 6 months, respectively).

Only 30% of MSM aged ≤ 26 years received three doses of HPV vaccine via the time-limited catch-up HPV vaccination program for MSM through a sexual health clinic, this is significantly lower than the completion rate of boys who received their HPV vaccines from the school-based program (i.e., 75.9% among boys turning 15 years in 2017) (12). The UK started a pilot HPV vaccination program for MSM aged ≤ 45 years in 2016-2018 via sexual health and HIV clinics and they found that the proportion of eligible MSM who received three doses of HPV vaccine was 27% (783/1677) via HIV clinics and 13% (10168/76033) via sexual health clinics, which is even lower than the estimate in our cohort (13). Similar to Australia, Canada is one of the countries that has implemented the gender-neutral school-based HPV vaccination program, and some provinces in Canada have also implemented a targeted MSM HPV vaccination program (8). A Canadian study reported that the vaccine completion among MSM aged ≤ 26 years was low (14% in Vancouver, 15% in Montreal and 21% in Toronto), and the vaccine completion was even lower among older MSM aged ≥27 years (9% in Vancouver, 2% in Montreal and 17% in Toronto) (8).

There are several factors found to be associated with HPV vaccine completion. First, we found that HIV-positive men are more likely to receive the full course of HPV vaccination compared to HIV-negative men. HIV-positive men are at a higher risk of acquiring anal cancer (14), and therefore, they may be more aware of HPV but they are also more engaged with healthcare services. The main reason for higher completion rate in HIV-positive men could be due to their regular attendance at HIV services at MSHC where they are reminded by clinicians to complete the subsequent doses. Second, we found that gay men are more likely to receive the full course of vaccine compared to bisexual men. It is likely that gay men could be more attached in the gay community; hence, they are more aware about their sexual health and HPV vaccination compared to bisexual men. Additionally, this is an important finding because gay men are less likely to receive herd protection from vaccinated women compared to bisexual men. Third, past studies have shown that past history of genital warts is associated with HPV vaccination initiation (9); however, there is no evidence suggesting there is an association between past history of genital warts and HPV vaccine completion.

A few limitations in this study should be noted. This study was conducted at a single urban sexual health clinic in Melbourne, and our findings may not be generalisable to other settings, particularly settings without a free HPV vaccination program for MSM. Additionally, low HPV vaccine completion rate in our study could be due to vaccination was administered opportunistically when men present with STI symptoms or STI testing compared to self-initiated vaccination at general practice clinic. Furthermore, our estimate on vaccine completion may be underestimated as men might have received the HPV vaccine via their general practices or immunisation providers outside MSHC; however, we were unable to justify this. Second, data on sexual practices such as the number of partners and condomless sex are self-reported; and thus, recall bias may have occurred.

Although the HPV vaccine completion rate among MSM aged 16–26 years via the time-limited catch-up HPV vaccination program was only 30%, it has been suggested that it may be cost-effective in reducing the burden of HPV diseases in modelling studies (15). A previous Australian-based model estimated that if 20% of unvaccinated young MSM receive the vaccine from a targeted MSM program each year, this may result in reducing low- and high-risk HPV infections by 71%, and anal cancer incidence by 23% by 2036 (15). However, it is important to note that most of these men receive the vaccine via the catch-up program might have already exposed to HPV before they receive the vaccine (4), which also may limit its effectiveness compared those who receive from the school-based program where boys receive their vaccine before sexual initiation. Further studies will be beneficial to understand the barriers to HPV vaccination that could increase the uptake as well as completion of HPV vaccination.

## Data Availability Statement

The datasets presented in this article are not readily available because data cannot be made publicly available in order to protect patient privacy as per the approved ethics requriment. Requests to access the datasets should be directed to Eric P.F. Chow, eric.chow@monash.edu.

## Ethics Statement

The studies involving human participants were reviewed and approved by Alfred Hospital Ethics Committee. Written informed consent from the participants' legal guardian/next of kin was not required to participate in this study in accordance with the national legislation and the institutional requirements.

## Author Contributions

EC conceived and designed the study. KH and RW performed chart review to verify the vaccination records. KH wrote the first draft of the manuscript. KH and ER performed literature review. KH and EC performed statistical analyses. All authors provided data interpretation, were involved in revising the manuscript for important intellectual content and approved the final version.

## Funding

CF and CB are supported by an Australian National Health and Medical Research Council (NHMRC) Leadership Investigator Grants (GNT1172900 and GNT1173361). EC is supported by an NHMRC Emerging Leadership Investigator Grant (GNT1172873).

## Conflict of Interest

EC, MC, and CF have received research funding from Merck Sharp & Dohme, outside the submitted work. EC has received an honorarium from Merck Sharp & Dohme, and Roche, outside the submitted work. The remaining authors declare that the research was conducted in the absence of any commercial or financial relationships that could be construed as a potential conflict of interest.

## Publisher's Note

All claims expressed in this article are solely those of the authors and do not necessarily represent those of their affiliated organizations, or those of the publisher, the editors and the reviewers. Any product that may be evaluated in this article, or claim that may be made by its manufacturer, is not guaranteed or endorsed by the publisher.
